# Appropriateness of Antibiotic Prescription for Prophylactic Purposes among Italian Dental Practitioners: Results from a Cross-Sectional Study

**DOI:** 10.3390/antibiotics10050547

**Published:** 2021-05-08

**Authors:** Aida Bianco, Vincenza Cautela, Francesco Napolitano, Francesca Licata, Maria Pavia

**Affiliations:** 1 Department of Health Sciences, School of Medicine, University of Catanzaro “Magna Græcia”, Viale Europa, 88100 Catanzaro, Italy; a.bianco@unicz.it (A.B.); cautvin@gmail.com (V.C.); francesca17licata@gmail.com (F.L.); 2 Department of Experimental Medicine, University of Campania ‘‘Luigi Vanvitelli”, Via L. Armanni, 5, 80138 Naples, Italy; francesco.napolitano2@unicampania.it

**Keywords:** antimicrobial resistance, antibiotic prescription, dentistry, prophylaxis, Italy

## Abstract

The primary objective of this study was to investigate the pattern of antibiotic prescription for prophylaxis purposes among Italian DPs (dental practitioners). A nationwide cross-sectional study was conducted using a multi-stage sampling design. A structured questionnaire was used to collect socio-demographic data and information about antibiotic prophylaxis (AP) prescriptions for selected dental diagnoses and surgical procedures. The presence of an indication and appropriateness of AP were defined according to international guidelines. In total, 563 DPs answered the questionnaire (response rate 52.6%). The proportions of DPs who prescribed AP in the presence of an indication ranged from 39.1% for luxation injury with soft tissue trauma to 73.1% for dental implants, whilst DPs who prescribed AP in healthy patients ranged from 41.9% in luxation injury with soft tissue trauma to 70.3% for bone grafting. The course of AP reported by DPs was not consistent with the guidelines in 70.9% of explored procedures. A high proportion of AP prescriptions before dental procedures were unnecessary. This highlights the urgent need to incorporate recommendations for best practices into national and local protocols as soon as they are established. Specific antibiotic stewardship strategies targeted to DPs should be implemented and assessed for effectiveness in improving prescribing of antibiotics.

## 1. Introduction

The global emergence and spread of antibiotic resistance (AR) is undermining the efficacy of one of the most powerful tools for fighting life-threatening infections. The threat of AR compromises progress in health care and life expectancy, and addressing this issue requires slowing the development of resistance through better antibiotic use in the first place. A number of organizations have initiated strategies to improve antibiotic utilization [1,2,3,4], and have launched several campaigns aimed at educating healthcare providers and consumers about appropriate antibiotic prescribing and use [5].

Dental practitioners (DPs) are among the top specialty prescribers of antibiotics, both for therapeutic and prophylaxis purposes [6], and antibiotics are among the most commonly prescribed drugs by DPs [7,8].

The rationale for prophylaxis in dental procedures is that high-risk patients, such as patients with a previous diagnosis of infective endocarditis (IE), patients with a prosthetic valve or with prosthetic material used for cardiac valve repair, or patients with replacement of joint prosthesis, have an increased risk for serious distant sites infections (e.g., IE and prosthetic joint infection (PJI)) secondary to bacteremia originating during dental care [9]. However, guidelines for antibiotic prophylaxis (AP) were revised to narrow the indications for prophylaxis in the 2007, 2013, and 2015 updates [10,11,12], due to a lack of evidence supporting an association between certain dental procedures (i.e., not requiring manipulation of the gingival or periapical region of teeth or perforation of the oral mucosa) and secondary infections even in high-risk patients, combined with increased awareness on the risk of antibiotic-associated adverse events [9].

While studies in outpatient primary medical care settings have demonstrated that from 30% [13] to 66.5% [14] of prescribed antibiotics are unnecessary, there are few data on the antibiotic-prescribing practices for prophylaxis purposes and its appropriateness among DPs. A survey of DPs found that 70% of them reported inappropriate prescription of prophylactic antibiotics prior to a dental procedure [15]. In a UK study of antibiotic prescribing among general dental practices, only 19% of antibiotics were prescribed in situations where their use was indicated by clinical guidelines [16]. A recent systematic review of the literature evaluated the protocol of AP adopted during third molar extraction and suggested evaluating the local and general health conditions of the patients before suggesting any drug prescription [17].

Therefore, the primary objective of this study was to investigate the pattern of antibiotic prescription for prophylaxis purposes among Italian DPs for selected surgical dental procedures. We hypothesized that AP would be frequently prescribed without any indication. Secondary objectives were to determine whether the prescriptions comply with the recommended guidelines in terms of drug choice, dose, timing, and duration.

## 2. Materials and Methods

### 2.1. Study Design

A nationwide cross-sectional study was conducted in a representative sample of the Italian general and specialist DPs. The information was collected through a mixed technique, which used an online questionnaire that was filled in by respondents, a direct telephone interview with the questionnaire administered by an interviewer, and a self-administered paper questionnaire.

### 2.2. Sample Size

The sample size was determined in order to warrant estimation of proportions with an expected margin of error of 5%, assuming an intended confidence interval (CI) of 95%. We used the prevalence of antibiotic prescription in dentistry obtained from similar studies [16,18]. Based on these assumptions, a sample of at least 500 DPs was required. The estimated sample size was inflated since previous similar surveys showed a non-participation rate between 50 and 70%, so that a total of 1250 DPs were invited to join the survey.

### 2.3. Survey Sampling Methods

To be eligible, DPs had to be registered within a Register of Physicians, Surgeons, and Dentists (RPSD). In Italy each physician, surgeon, or dentist who practices the profession has to register for the local RPSDs. DPs not having a valid e-mail/telephone contact, not practicing dental care, or having moved abroad were not included in the survey.

To obtain a representative sample of the Italian DPs, a multi-stage sampling design was used. First, the whole country was divided into five areas (North-West, North-East, Center, South, and Islands) according to the geographical division by the Italian National Institute of Statistics (ISTAT) [19]. In each area, a random sample of five RPSDs was selected from a publicly available frame of all RPSDs. The aims of the study were delineated to the chief executive of each local RSPD by telephone, and at the same time verbal consent was obtained. In case permission was refused, we randomly chose another RPSD in the same area, and so forth, until consent was given. Next, 50 DPs were randomly chosen among those registered within each selected RPSD, to give a total sample of 1250 DPs. Enrolled DPs were sent recruitment e-mails containing a link directing them to the home page of the online survey where they were invited to provide anonymous responses to a self-administered web-questionnaire, pre-tested on a convenience sample of DPs. Non-responders were repeatedly contacted by three e-mail reminders. In an attempt to maximize the response rate, a telephone call was made to the DPs in case they preferred to give their answers via a telephone interview. Moreover, some RSPD preferred to distribute a paper questionnaire to the DPs.

Potential participants were informed at the beginning of the questionnaire on the purpose of the study, and on its voluntary nature, together with the condition that they could terminate their participation at any stage of the survey.

### 2.4. Instruments and Methods for Data Collection

Data collection was performed between 1 April and 30 October 2019. A structured questionnaire was used, comprising four sections: (1) an introductory section presenting study aims and motivation at the end of which DPs could give their written consent in joining the study, (2) a socio-demographic data collection section, (3) the main research section about practices regarding prophylactic antibiotic prescriptions, (4) and sources of information used to update knowledge on antibiotic use in dental practices.

The web questionnaire was developed after an extensive literature review [20,21,22,23] and contained open-ended and close-ended questions. We explored DPs’ prescription habits for the following dental diagnoses and surgical procedures: traumatic dental injuries (i.e., luxation injury with soft tissue trauma and luxation injuries of the permanent dentition), third molar extraction, dental implants, bone grafting, and replantation of avulsed permanent teeth. For each diagnosis or surgical procedure, DPs were asked if they usually prescribed an antibiotic for prophylaxis purposes (close-ended questions with a multi-option response format). Details regarding drug choice (open-ended question), timing, and duration of AP (close-ended questions with a multi-option response format) were also collected for each diagnosis or surgical procedure. Moreover, DPs were asked if the information about clinical history and other concomitant treatments was usually investigated, and if they usually advised patients about antibiotic regimen (eight items, closed-ended questions with a “yes/no” response format). A copy of the questionnaire is reported as a Supplementary Material. AP indication was defined according to international guidelines [9,11,12,24,25,26,27,28,29,30]. Briefly, we considered AP was indicated before all the procedures that involve manipulation of the gingival or periapical region of the teeth or perforation of the oral mucosa (i.e., luxation injury with soft tissue trauma and all above-mentioned surgical procedures) in high-risk patients. In accordance with published guidelines, we considered high-risk patients those with a previous diagnosis of IE, patients with a prosthetic valve or with prosthetic material used for cardiac valve repair, and patients with replacement of joint prosthesis in the previous 6 months. Regarding the antibiotic regimen, oral prescription of two grams of amoxicillin between 30 min and 1 h prior to procedure, in a single dose, was considered appropriate [10,28]. In penicillin-allergic patients, the correct antibiotic regimen was oral prescription of 600 mg of clindamycin, in accordance with recommendations [28].

Ethical approval was granted by the local Human Research Ethics Committee (ID No. 121/2019/04/18).

### 2.5. Statistical Analysis

Statistical analysis was performed using STATA software program, version 16 [31]. Data were summarized using frequencies for categorical data and means and standard deviations (SDs) for continuous data. In the first stage of the inferential analysis, a simple binomial analysis was carried out to evaluate the effect of the independent variables on the DP’s correct prescription practice (0 = AP prescription by DPs without an indication and no AP prescription by DPs with an indication; 1 = AP prescription by DPs with an indication and no AP prescription by DPs without an indication). In the second stage, a multilevel mixed-effects logistic regression analysis was conducted to examine the potential association of correct prescription practice with the following explanatory variables: gender, age, residence, number of years in practice, post degree specialization, indication for AP, and type of surgical procedure. The interaction between the surgical procedures and the presence of indication or not, was also investigated. With the aim of accounting for the multilevel dataset structure (procedures are “nested” within DPs), the variable DP ID was introduced in the model as random factor. A significance level of 5% was used for hypothesis testing. Adjusted odds ratio and 95% confidence intervals were calculated.

## 3. Results

Of the 1250 selected DPs invited to participate in the study, 81 were ineligible because they did not practice dental care or had moved abroad, and 98 were not included because of incorrect e-mail/telephone contact information; 563 answered the general questionnaire, giving a response rate of 52.6%. The majority of respondents were males (71.2%) and the mean age was 50.6 years (range 25–77 years). The most frequent degree was DDS (60.9%) and the mean number of years in practice was 25 (SD ± 10.7). Almost one third (31.2%) of the sample had a post-degree specialization (20.8% in oral surgery, 18.8% in stomatology. and 11.9% in orthodontics). Of the DPs, 34.8% were from the North-West area, 30.7% from the South, 13.7% from the Center, 13.3% from North-East, and 7.5% from the Islands. The vast majority of DPs reported informing the patient about the AP regimen (99.6%), the importance of correct dosage (99.8%) and duration (99.8%), and the possible consequences of non-adherence to therapy (87.4%). Similarly, a high proportion of DPs reported asking their patients for previous high-risk conditions, such as IE (84%), prosthetic cardiac valves (93.6%), and complex congenital heart defects (97.6%).

The AP prescription in the explored dental procedures, according to presence or absence of an indication, is shown in Table 1. For the explored dental procedures, the proportions of DPs who prescribed AP in the presence of an indication ranged from 39.1% for luxation injury with soft tissue trauma to 73.1% in dental implants, whilst DPs who were used to prescribe AP in healthy patients, i.e., without a clinical indication, ranged from 41.9% in luxation injury with soft tissue trauma to 70.3% for bone grafting. Prescription of an antibiotic was never indicated by the guidelines in luxation injury of the permanent dentition and it was prescribed in 17.4% of cases. Overall, guideline-concordant prescribing of AP (i.e., prescribing with indication and not prescribing without indication) ranged from 35.6% for luxation injuries with soft tissue trauma to 55.2% for dental implants.

The AP prescription approach in procedures with indication according to drug choice, timing, and duration is presented in Table 2. Amoxicillin and clavulanate was the most frequently prescribed antibiotic in all explored dental procedures in patients who did not report penicillin allergy, ranging from 51.8% in luxation injury with soft tissue trauma to 61.3% in bone grafting. Amoxicillin was less frequently prescribed than amoxicillin and clavulanate, ranging from 33.1% in bone grafting to 38.6% in luxation injury with soft tissue trauma. A high proportion of DPs (77.2%) self-reported clindamycin (600 mg) prescription in penicillin allergic patients, in accordance with recommendations. Adherence to all components of AP (drug choice, timing, and duration) ranged from 5.2% in luxation injury with soft tissue trauma to 9.6% in dental implant. AP was inappropriately prolonged over 24 h in the great majority of cases, ranging from 81.8% in third molar extraction and luxation injury with soft tissue trauma to 85.7% in replantation of avulsed permanent teeth. Adherence to timing was respected in almost three quarters of the procedures, and in the remaining cases AP was prescribed even 48 h prior to the procedure.

The course of AP reported by DPs was not consistent with the guidelines in 70.9% of explored procedures with one or more reasons for inappropriateness. The reasons for the inappropriate prescription were prolonged duration (83.2%) with an average of 7.0 ± 1.0 day, with the majority prescribing for 6–8 days, timing (39.7%), and use of a broad-spectrum molecule (18.6%).

Continuing education courses were the main sources of information about AP in dentistry (43.7%), followed by scientific journals and academic societies (42.8%). Two-thirds of respondents were aware of available guidelines/recommendations for prescribing of AP in dentistry, and 82.1% of the DPs reported an interest in more education to improve their knowledge and practices about AP in dentistry.

The simple binomial logistic regression analyses did not show any statistically significant difference between the correct prescription practice and the DPs’ socio-demographic or professional characteristics.

The multilevel mixed-effect logistic model showed that none of the socio-demographic and professional characteristics were associated with the correct prescription practice, excluding being a DP practicing in the Central Italy. Indeed those DPs had higher odds (OR = 1.55, CI 95% = 1.04–2.32) of correct prescription practice compared with those of Southern Italy. Regarding the invasive dental procedures and the presence of indication or not, an overall statistically significant gain of a correct prescription practice (OR = 5.1, CI 95% = 3.79–6.86) was shown when an indication of prescription was present. Moreover, a significant interaction was observed. Indeed, the correct prescription practice in patients with luxation injuries (compared with reference category) was less likely when an indication was present than when it was not (OR = 0.29, CI 95% = 0.19–0.44). On the contrary, the odds of correct prescription in cases of dental implant (OR = 1.92, CI 95% = 1.25–2.95) and replantation of avulsed permanent teeth (OR = 1.50, CI 95% = 1.12–2.01) were higher (compared with reference category) when indication was present. Finally, 31% of the model residual variability was explained by the physician random factor (intraclass correlation = 0.31, CI 95% = 0.26–0.35) (Table 3).

## 4. Discussion

To the best of our knowledge, this study represents the first national evaluation of prophylactic antibiotic prescribing for two groups of common dental procedures (i.e., traumatic dental injuries and surgical invasive procedures), for which antibiotics are currently being prescribed in the absence of an indication. Our goal was to identify opportunities to improve AP prescribing through future tailored interventions.

This survey has provided four major findings. First, our results demonstrate that 84.2% of the surveyed DPs reported prescribing AP in accordance with guidelines recommendations (i.e., procedures with manipulation of gingival tissue or the periapical region of the teeth in high-risk patients). Conversely, it is astonishing that a similar proportion of DPs (72.3%) prescribed AP for dental procedures in ordinary conditions (i.e., healthy patients) and, therefore, without indication. In addition with these figures, the finding that the correct prescription practice was significantly more likely when the prescription was indicated suggests that DPs are not always aware of the current clinical guidelines regarding antibiotic prophylaxis, even though guidelines are available, and, when in doubt, prefer to prescribe [14,32]. It should be highlighted, however, that the issue of when and for what dental conditions systemic AP is necessary is still controversial, even in the case of IE and PJIs where evidence-based guidelines to guide DPs in clinical practice are available [9,11,12,24,25,26,27,28]. 

The second key result is that the explored surgical invasive procedures, including impacted third molars and implant surgery, were routinely covered by prescription of systemic AP without an indication. It could be argued that antibiotics administered prior to a number of dental surgical procedures, such as impacted third molars and implant surgery, were also prescribed to prevent postoperative local complication, such as the surgical site infection (SSI), despite the fact that evidence on the effectiveness of prophylactic antibiotics to prevent SSIs in the mouth is from poor to non-existent [22]. Indeed, a systematic review and meta-analysis evaluating AP in dental implant and extraction procedures indicated that peri-operative routine parenteral AP in healthy patients is not required or recommended, and suggested that clinicians must carefully consider the use of antibiotics due to the risk of the development of AR [33]. In contrast, a recent Cochrane Systematic Review with a meta-analysis including six trials [34] suggested that administration of a single preoperative dose of amoxicillin significantly reduces early failure of dental implants placed in ordinary conditions (healthy patients). The rationale is that infections around biomaterials are difficult to treat, and almost all infected implant have to be removed.

Thirdly, most participants consistently prescribed various types of antibiotics and prophylactic regimens without any evidence-based support. Regarding the choice of drug, the reported practice was not well aligned with the guidelines for patients with no allergy to penicillin. Indeed, more than half of DPs prescribed amoxicillin and clavulanate instead of amoxicillin, the option of first choice in all the explored dental procedures [9,11,12,24,25,26,27,28,29,30]. Narrow spectrum antibiotics should always be considered the first choice for AP purpose; while broad spectrum antibiotics play an invaluable role in the treatment of bacterial infections, there are some drawbacks to their prophylactic use, namely selection for and spread of resistance across multiple bacterial species, and the detrimental effect they can have upon the host microbiome [35]. Another opportunity for improvement is related to the timing and duration of AP; 23.1% of DPs prescribed it one or two days before the procedure and 83.1% until 7 days after. When indicated, the AP has to be timely administrated (30–60 min preoperatively), and should not be continued after the procedure. Previous studies demonstrated that timing of AP was an important opportunity for improvement [36,37] since longer durations have not shown to be more beneficial, and this practice may result in the selection of resistant strains.

Fourthly, in almost 60% of luxation injuries with soft tissue trauma, although a prescription was indicated, no AP was prescribed, and the correct prescription practice was less likely when an indication was present than when it was not, resulting in the antibiotic under-prescription. Regarding management of luxation injuries, it should be pointed out that the consensus statement of the International Association of Dental Traumatology has stated that limited evidence exists for the use of systemic antibiotics, and antibiotic use remains at the discretion of DP in presence of a soft tissue trauma [29,30]. Additionally, the European Society of Cardiology recommends AP for the prevention of IE in the highest-risk patients when both a luxation injury and a perforation of the oral mucosa occur [28].

Taken together, the findings of this study demonstrated that evidence-based recommendations on AP provided by guidelines are not consistently followed by DPs. Moreover, the results suggest that the inappropriate use of AP may be related to limited awareness of guidelines, as well as to misunderstanding generated by rapidly updated and sometimes controversial guidelines, and indeed the vast majority of DPs (82.1%) perceived the need for additional information regarding AP in dentistry. Therefore, to improve the awareness of DPs of their role in restraining the increasing incidence of AR, it is imperative to harmonize the guidelines issued by different scientific societies in order to clearly represent the best available evidence and to put in place efforts for establishing an effective antimicrobial stewardship in dentistry.

### Strengths and Limitations

The national level of the study participants and the large sample size represent key strengths of this survey.

To appreciate the findings of this cross-sectional study, some potential limitations in the design and measurements need to be considered. First, the validity of the findings reported in this article is limited by self-reporting and is subject to bias. Intentional deception, poor recall, or misunderstanding of the questions can all contribute to a wrong assumption of actual prescribing behavior. DPs might report socially acceptable responses that are different from the actual day-to-day practices. However, retrospective manual medical record review of AP prescribing was considered impracticable since accurate national administrative data are not available, as well as direct observation, because of expenses and because it may also erringly influence behavior. Nevertheless, it has been demonstrated that the means for improving the validity of self-reported data should include adherence to procedures that maximize anonymity and confidentiality, as we performed in the survey. Second, the fact that simple binomial and multilevel mixed-effects logistic analyses showed no statistically significant difference between the study groups could be due to inadequate statistical power to detect a meaningful difference. However, this was not our goal since we wanted only to assess DPs’ evidence-based practices related to prescription of AP. Third, it is well known that a desirable response rate must be higher than 60%; we believe that our response rate (52.6%) is satisfactory considering that DPs are a group with very low survey response rates [38,39,40,41] and that our response rate was similar to or higher than the 27.2–32% reported in analogous surveys [18,42,43,44].

## 5. Conclusions

The study findings showed that a high proportion of AP prescriptions before dental procedures are unnecessary. This worrisome scenario where DPs seemed unaware that these guidelines had been updated or that a new guidance procedure had been promulgated, highlights the urgency to adapt and incorporate recommendations of best practice into national and local protocols/guidelines as soon as they are established. Moreover, the empirical and broad use of AP reported is clearly no longer acceptable, and specific antibiotic stewardship strategies and prescribing tools targeted to DPs should be developed, implemented, and assessed for effectiveness in improving prescribing of antibiotics for infection prophylaxis.

## Figures and Tables

**Table 1:** Self-reported AP prescription in the explored dental procedures according to presence of an indication.

Dental Procedures	Appropriate AP Prescription ^1^ (563)		AP Over-Prescription ^2^ (563)		Total Prescription (1126) Guidelines Concordant AP	
	N	%	N	%	N	%
**Invasive procedures**						
Dental implant	423	73.1	365	64.8	621	55.2
Third molar extraction	330	58.6	389	69.1	504	44.8
Bone grafting	344	61.1	396	70.3	511	45.4
Replantation of avulsed permanent teeth	350	62.2	364	64.7	549	48.8
Luxation injuries with soft tissue trauma	220	39.1	382	41.9	401	35.6
**Non-invasive procedures ^3^**						
Luxation injuries of the permanent dentition	n/a		98	17.4	465 ^4^	82.6

AP, antibiotic prophylaxis. ^1^ AP prescription in high-risk patients (i.e., history of IE, prosthetic cardiac valves, or prosthetic joint replacement in the previous 6 months). ^2^ AP prescription in healthy patients. ^3^ Procedures without manipulation of the gingival or periapical region of the teeth or perforation of the oral mucosa in which AP is never indicated. ^4^ Eligible procedures.

**Table 2:** AP prescription approach in procedures with indication, according to drug choice, timing, and duration.

	Dental Implant		Third Molar Extraction		Bone Grafting		Replantation of Avulsed Permanent Teeth		Luxation Injury with Soft Tissue Trauma	
	N	%	N	%	N	%	N	%	N	%
**Drug choice**										
Appropriate (amoxicillin 2 g, single dose)	150	35.5	119	36.1	114	33.1	121	34.6	85	38.6
Inappropriate (unsuitable choice for AP)										
Amoxicillin and Clavulanate	251	59.1	194	58.8	211	61.3	209	59.7	114	51.8
Other ^1^	22	5.4	17	5.1	19	5.6	20	5.7	21	9.6
**Alternative Drug Choice ^2^**										
Appropriate (clindamycin 600 mg)	240	77.9	191	75.2	188	76.7	208	80	119	75.3
Inappropriate	68	22.1	63	24.8	57	23.3	52	20	39	24.7
**Timing**										
Appropriate (30–60 min prior to the procedure)	323	76.4	288	77.4	273	78.7	266	76	173	75.9
Inappropriate (24–48 h prior to the procedure)	100	23.6	84	22.6	74	21.3	84	24	55	24.1
**Duration**										
Appropriate (within 24 h from the procedure)	73	16.5	71	18.2	62	17	52	14.3	43	18.2
Inappropriate (over 24 h from the procedure)										
1–3 days	177	40	135	34.6	127	34.9	164	45.2	97	41.1
4–6 days	187	42.1	179	45.9	170	46.7	141	38.8	93	39.4
>6 days	6	1.4	5	1.3	5	1.4	6	1.7	3	1.3
**Appropriate drug choice, timing, and duration**	54	9.6	44	7.8	45	8	41	7.3	29	5.2

^1^ Includes nine different molecules (azithromycin, amoxicillin/metronidazole, bacampicillin hydrochloride, clarithromycin, ceftriaxone, clindamycin, lincomycin, miocamycin, and spiramycin). ^2^ In patients with penicillin allergy.

**Table 3:** Multilevel mixed-effect logistic regression model results exploring determinants of correct prescription practices.

Variable	OR	95% CI	*p*
**Outcome: Correct prescription practice** **Log likelihood = −2962.65; χ² = 591.71 (5 df); *p* ≤ 0.0001, No. of observations = 5060**			
**Age (years), continuous**	0.99	0.97–1.01	0.313
**Gender**			
Male ^1^	1.00		
Female	0.89	0.68–1.18	0.416
**Number of years in practice, continuous**	1.01	0.98–1.03	0.864
**Post-degree specialization**			
No ^1^	1.00		
Yes	1.09	0.82–1.43	0.552
**Residence**			
South ^1^	1.00		
Islands	0.77	0.41–1.46	0.427
Center	1.55	1.04–2.32	0.033
North-East	1.16	0.77–1.75	0.485
North-West	0.92	0.67–1.25	0.586
**Invasive dental procedures**			
Third molar extraction ^1^	1.00		
Luxation injuries with soft tissue trauma	1.19	0.88–1.60	0.255
Dental implant	1.45	1.08–1.95	0.014
Replantation of avulsed permanent teeth	1.50	1.12–2.01	0.007
Bone grafting	0.89	0.65–1.20	0.438
**Indication for A** **P**	5.1	3.79–6.86	<0.001
**Invasive dental procedures with indication**			
Third molar extraction with indication ^1^	1.00		
Luxation injuries with soft tissue trauma with indication	0.29	0.19–0.44	<0.001
Dental implant with indication	1.92	1.25–2.95	0.003
Replantation of avulsed permanent teeth with indication	0.81	0.54–1.22	0.315
Bone grafting with indication	1.27	0.84–1.93	0.265

^1^ Reference category.

## Data Availability

The data presented in this study are openly available in the Mendeley data repository (doi:10.17632/gbvhzbjnsm.1).

## Supplementary Materials

The following are available online at https://www.mdpi.com/article/10.3390/antibiotics10050547/s1.
