# Supplementary material for: Appropriateness of Antibiotic Prescription for Prophylactic Purposes among Italian Dental Practitioners: Results from a Cross-Sectional Study
Source: Antibiotics (Basel). 2021 May 8;10(5):547. doi: 10.3390/antibiotics10050547 (PMC8150285; doi:10.3390/antibiotics10050547)
Supplement: Supplementary file 1 [file antibiotics-10-00547-s001.zip › antibiotics-1161839-supplementary.pdf]

antibiotics

# DEPARTEMENT OF HEALTH SCIENCES

# MEDICAL SCHOOL

# UNIVERSITY OF CATANZARO "MAGNA GRÆCIA"

## Appropriateness of antibiotic prescription for prophylactic purposes among Italian dental practitioners

The phenomenon of antibiotic resistance is constantly increasing and among the most effective measures to reduce this problem is the conscious and appropriate use of antibiotics, both for prophylactic and therapeutic purposes. The aim of this study is to evaluate the prescriptive pattern of antibiotics in dentistry. The questionnaire is intended for Oral/Dental Surgeons, Orthodontists and Periodontists, Dental (Maxillofacial) and Cosmetic Surgeons, Dentists and Paediatric dentists and is divided into three sections: the participant's characteristics, practices regarding antibiotic prescription, and finally, sources of information. It should only take about 10 minutes. According to European Legislative Decree 679/2016 General Data Protection Regulation (GDPR) and Legislative Decree n.101/18, data will be analyzed only in an aggregate way and there will be no nominal data spreading. Precautions have been taken in order to guarantee confidentiality of gathered data and anonymity of respondents, so one can feel completely free to respond. Filling in the questionnaire implicitly entails the approval for the use of data for the purposes of this survey. Thank you for considering taking part in this research. We hope that you will help us with your participation in order to ensure a successful project.

### A. DEMOGRAPHIC AND PRACTICE CHARACTERISTICS

*A1.* YEAR OF BIRTH ____________________________ *A2.* GENDERM ❒ F ❒

*A3.* WHAT IS THE HIGHEST LEVEL OF EDUCATION THAT YOU HAVE COMPLETED?

Medical degree with ❒ or without ❒ specialty in dentistry

Degree in dentistry with ❒ or without ❒ specialty in (please specify)_______________________________________________

*A4*. WHERE DO YOU CURRENTLY LIVE?*(please indicate the town)* ________________________________

*A5.* HOW LONG HAVE YOU WORKED IN A DENTAL PRACTICE? *(please specify the number of years)*__________________________________

### B. PATTERN OF ANTIBIOTIC PRESCRIPTION FOR PROPHYLACTIC PURPOSES

*B1.* PLEASE, INDICATE IN THE CASE OF **REPLANTATION OF AVULSED PERMANENT TEETH** IN WHICH OF THE FOLLOWING PATIENTS DO YOU USUALLY PRESCRIBE AN ANTIBIOTIC

❒ healthy patients ❒ patients with infective endocarditis

❒ patients with prosthetic cardiac valves ❒ patients with prosthetic joint replacement in the previous 6 months

❒ none (*skip to* **B4**)

*B2.* PLEASE, SPECIFY THE TYPE (MOLECULE) AND THE DOSAGE OF ANTIBIOTIC THAT YOU PRESCRIBE TO A PATIENT NOT ALLERGIC TO PENICILLIN/AMOXICILLIN: ________________________________________

*B3.* PLEASE, INDICATE THE ANTIBIOTIC REGIMEN (more than one answer allowed):

❒ Single dose 30 minutes prior to the procedure ❒ Single dose 1 hour prior to the procedure

❒ Single dose in 24 hours prior to the procedure ❒ Single dose daily for 2 days prior to the procedure

❒ After procedures (indicate for how many days) 1 ❒ 2 ❒ 3 ❒ 4 ❒ 5 ❒ 6 ❒ 7 ❒ ≥ 8 ❒

*B4.* PLEASE, INDICATE IN THE CASE OF **DENTAL IMPLANT** IN WHICH OF THE FOLLOWING PATIENTS DO YOU USUALLY PRESCRIBE AN ANTIBIOTIC

❒ healthy patients ❒ patients with infective endocarditis

❒ patients with prosthetic cardiac valves ❒ patients with prosthetic joint replacement in the previous 6 months

❒ none (*skip to* **B7**)

*B5.* PLEASE, SPECIFY THE TYPE (MOLECULE) AND THE DOSAGE OF ANTIBIOTIC THAT YOU PRESCRIBE TO A PATIENT NOT ALLERGIC TO PENICILLIN/AMOXICILLIN: ________________________________________

*B6.* PLEASE, INDICATE THE ANTIBIOTIC REGIMEN:

❒ Single dose 30 minutes prior to the procedure ❒ Single dose 1 hour prior to the procedure

❒ Single dose in 24 hours prior to the procedure ❒ Single dose daily for 2 days prior to the procedure

❒ After procedures (indicate for how many days) 1 ❒ 2 ❒ 3 ❒ 4 ❒ 5 ❒ 6 ❒ 7 ❒ ≥ 8 ❒

*B7.* PLEASE, INDICATE IN THE CASE OF **LUXATION INJURIES WITH SOFT TISSUE TRAUMA** IN WHICH OF THE FOLLOWING PATIENTS DO YOU USUALLY PRESCRIBE AN ANTIBIOTIC

❒ healthy patients ❒ patients with infective endocarditis

❒ patients with prosthetic cardiac valves ❒ patients with prosthetic joint replacement in the previous 6 months

❒ none (*skip to* **B10**)

*B8.* . PLEASE, SPECIFY THE TYPE (MOLECULE) AND THE DOSAGE OF ANTIBIOTIC THAT YOU PRESCRIBE TO A PATIENT NOT ALLERGIC TO PENICILLIN/AMOXICILLIN: ________________________________________

*B9.* PLEASE, INDICATE THE ANTIBIOTIC REGIMEN:

❒ Single dose 30 minutes prior to the procedure ❒ Single dose 1 hour prior to the procedure

❒ Single dose in 24 hours prior to the procedure ❒ Single dose daily for 2 days prior to the procedure

❒ After procedures (indicate for how many days) 1 ❒ 2 ❒ 3 ❒ 4 ❒ 5 ❒ 6 ❒ 7 ❒ ≥ 8 ❒

***B10.*** PLEASE, INDICATE IN THE CASE OF **BONE GRAFTING** IN WHICH OF THE FOLLOWING PATIENTS DO YOU USUALLY PRESCRIBE AN ANTIBIOTIC

❒ healthy patients ❒ patients with infective endocarditis

❒ patients with prosthetic cardiac valves ❒ patients with prosthetic joint replacement in the previous 6 months

❒ none (*skip to* **B13**)

***B11.*** PLEASE, SPECIFY THE TYPE (MOLECULE) AND THE DOSAGE OF ANTIBIOTIC THAT YOU PRESCRIBE TO A PATIENT NOT ALLERGIC TO PENICILLIN/AMOXICILLIN: ________________________________________

***B12.*** PLEASE, INDICATE THE ANTIBIOTIC REGIMEN:

❒ Single dose 30 minutes prior to the procedure ❒ Single dose 1 hour prior to the procedure

❒ Single dose in 24 hours prior to the procedure ❒ Single dose daily for 2 days prior to the procedure

❒ After procedures (indicate for how many days) 1 ❒ 2 ❒ 3 ❒ 4 ❒ 5 ❒ 6 ❒ 7 ❒ ≥ 8 ❒

***B13.*** PLEASE, INDICATE IN THE CASE OF **THIRD MOLAR EXTRACTION** IN WHICH OF THE FOLLOWING PATIENTS DO YOU USUALLY PRESCRIBE AN ANTIBIOTIC

❒ healthy patients ❒ patients with infective endocarditis

❒ patients with prosthetic cardiac valves ❒ patients with prosthetic joint replacement in the previous 6 months

❒ none (*skip to* **B16**)

***B14.*** PLEASE, SPECIFY THE TYPE (MOLECULE) AND THE DOSAGE OF ANTIBIOTIC THAT YOU PRESCRIBE TO A PATIENT NOT ALLERGIC TO PENICILLIN/AMOXICILLIN: ________________________________________

***B15*** PLEASE, INDICATE THE ANTIBIOTIC REGIMEN:

❒ Single dose 30 minutes prior to the procedure ❒ Single dose 1 hour prior to the procedure

❒ Single dose in 24 hours prior to the procedure ❒ Single dose daily for 2 days prior to the procedure

❒ After procedures (indicate for how many days) 1 ❒ 2 ❒ 3 ❒ 4 ❒ 5 ❒ 6 ❒ 7 ❒ ≥ 8 ❒

***B16.*** PLEASE, INDICATE IN THE CASE OF **LUXATION INJURIES OF THE PERMANENT DENTITION** IN WHICH OF THE FOLLOWING PATIENTS DO YOU USUALLY PRESCRIBE AN ANTIBIOTIC

❒ healthy patients ❒ patients with infective endocarditis

❒ patients with prosthetic cardiac valves ❒ patients with prosthetic joint replacement in the previous 6 months

❒ none (*skip to* **B19**)

***B17.*** PLEASE, SPECIFY THE TYPE (MOLECULE) AND THE DOSAGE OF ANTIBIOTIC THAT YOU PRESCRIBE TO A PATIENT NOT ALLERGIC TO PENICILLIN/AMOXICILLIN: ________________________________________

***B18.*** PLEASE, INDICATE THE ANTIBIOTIC REGIMEN:

❐ Single dose 30 minutes prior to the procedure ❐ Single dose 1 hour prior to the procedure

❐ Single dose in 24 hours prior to the procedure ❐ Single dose daily for 2 days prior to the procedure

❐ After procedures (indicate for how many days) 1 ❐ 2 ❐ 3 ❐ 4 ❐ 5 ❐ 6 ❐ 7

***B19.*** IN CASES WHEN A PATIENT IS ALLERGIC TO PENICILLIN, WHICH TYPE (MOLECULE) AND DOSAGE OF ANTIBIOTIC DO YOU PRESCRIBE?

▪Clindamycin 600mg ❐ 300mg ❐ ▪Metronidazole 1g ❐ 500mg ❐

▪ Other (specify type of antibiotic/molecule and dosage) ________________________________

***B20.*** DURING CLINICAL PRACTICE, DO YOU ADVICE YOUR PATIENT ABOUT:

| | |
|---|---|
| *ways of taking an antibiotic prescription?* | *Yes* ❐ *No* ❐ |
| *antibiotic dosage?* | *Yes* ❐ *No* ❐ |
| *antibiotic duration?* | *Yes* ❐ *No* ❐ |
| *possible consequences of non-adherence to antibiotic prescription?* | *Yes* ❐ *No* ❐ |

***B21.*** DO YOU ASK YOUR PATIENT IF HE/SHE HAS:

| | |
|---|---|
| *a history of infective endocarditis?* | *Yes* ❐ *No* ❐ |
| *prosthetic cardiac valves?* | *Yes* ❐ *No* ❐ |
| *taken a course of antibiotics in the last week before prescribing antibiotics for dental procedures?* | *Yes* ❐ *No* ❐ |
| *had a prosthetic joint replacement in the previous 6 months?* | *Yes* ❐ *No* ❐ |

**C. SOURCES OF INFORMATION**

***C1.*** ARE YOU AWARE OF ANY AVAILABLE GUIDELINES/RECOMMENDATIONS ON THE USE OF SYSTEMIC ANTIBIOTICS FOR PROPHYLACTIC PURPOSES IN DENTISTRY? ❐ *No (skip to C3)* ❐ *Yes*

*(Please indicate the guidelines that you are aware of)*

________________________________________________________________________________________

________________________________________________________________________________________

***C2.*** DO YOU FOLLOW THESE GUIDELINES? *Yes* ❒ *No* ❒

***C3.*** WHICH OF THE FOLLOWING ARE YOUR SOURCES OF INFORMATION?

*None* ❒ *Internet* ❒ *Mass Media* ❒ *Scientific Journals* ❒ *Senior colleagues* ❒ *Corporate training courses* ❒

*Scientific societies* ❒ *Other (Please specify)*________________________________________

***C4.*** DO YOU THINK YOU NEED FURTHER INFORMATION ABOUT THE USE OF SYSTEMIC ANTIBIOTICS FOR PROPHYLACTIC PURPOSES IN DENTISTRY? *Yes* ❒ *No* ❒

*If you would like to add something more, please write it down in the space below*

________________________________________________________________________________________

________________________________________________________________________________________

________________________________________________________________________________________

*Thank you for answering our questionnaire.*
